# Supplementary material for: Cryo-EM structures of HIV-1 trimer bound to CD4-mimetics BNM-III-170 and M48U1 adopt a CD4-bound open conformation
Source: Nat Commun. 2021 Mar 29;12:1950. doi: 10.1038/s41467-021-21816-x (PMC8007822; doi:10.1038/s41467-021-21816-x)
Supplement: Supplementary file 1 — Supplementary Information [file 41467_2021_21816_MOESM1_ESM.pdf]

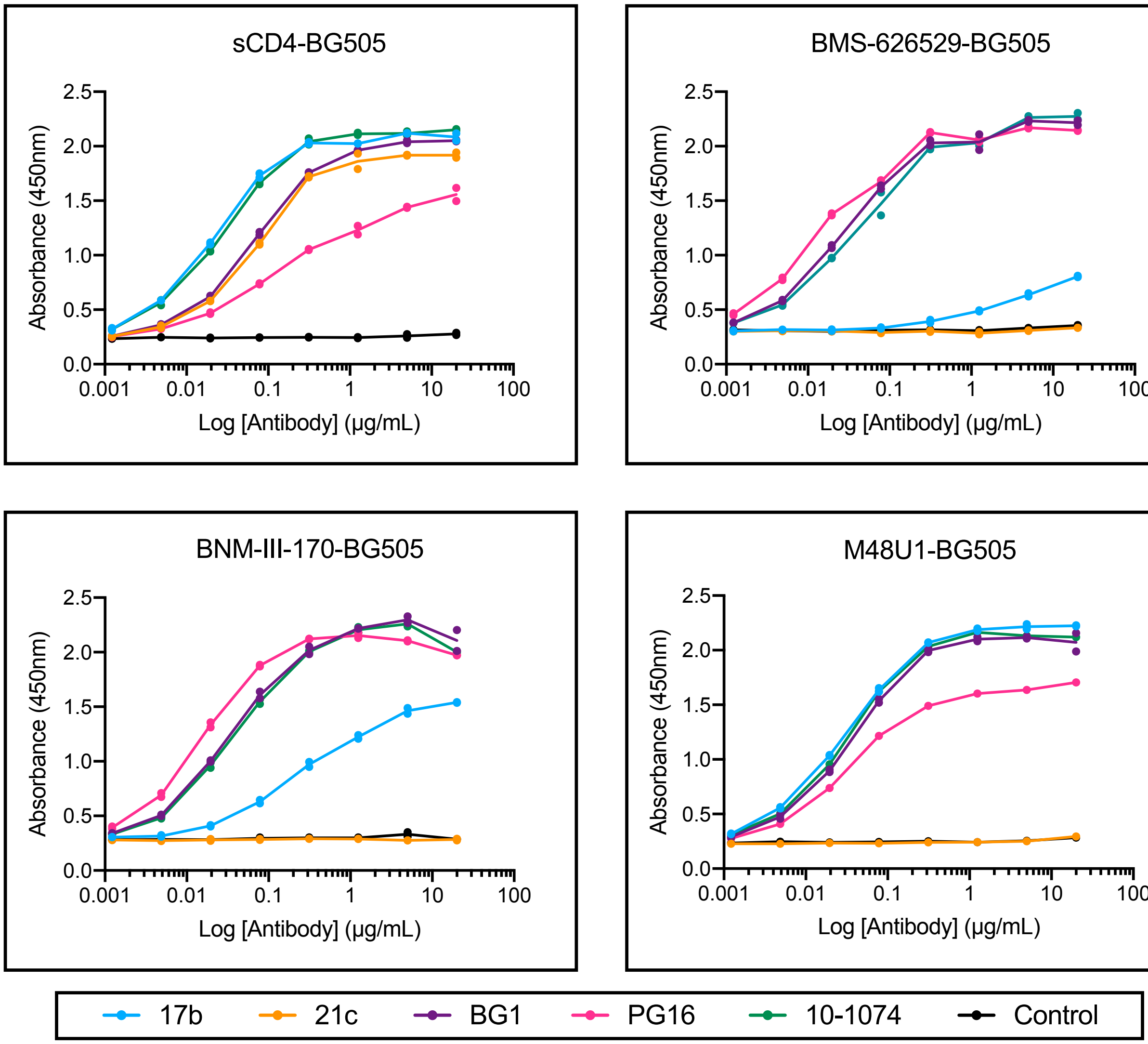

Supplementary Figure 1. Binding of HIV-1 antibodies to sCD4-Env or CD4m-Env complexes.

ELISAs evaluating binding of IgG versions of anti-Env antibodies 17b, 21c, BG1, PG16, and 10-1074 or control (no antibody added) to **a**, sCD4-BG505 Env, **b**, BMS-626529-BG505, **c**, BNM-III-170-BG505 Env, and **d**, M48U1-BG505 Env. Values are shown as mean of two individual replicates. Colors: 17b = light blue, 21c = light orange, BG1 = purple, PG16 = hot pink, 10-1074 = green, control = black. Results shown for n=2 individual replicates. Connecting line is for mean of individual replicates.

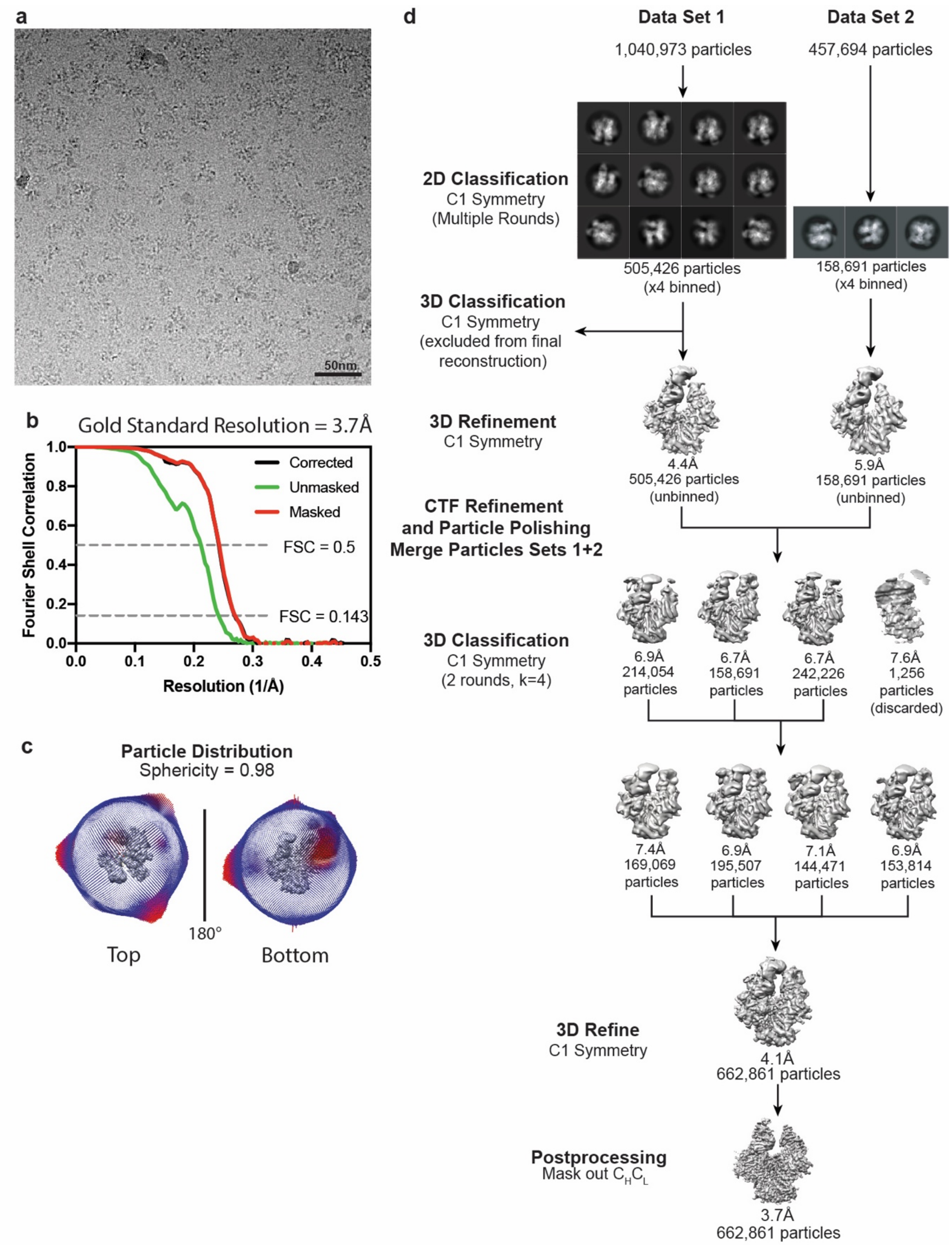

Supplementary Figure 2. Data Processing for BNM-III-170-BG505-17b complex.
a, Representative EM micrograph of data set. 2739 and 2048 micrographs were collected for data set 1 and 2, respectively. Scale bar is 50nm. **b,** Gold Standard 3D FSC chart for final reconstruction map using combined data sets. **c,** Particle orientation distribution and sphericity for final reconstruction of combined data sets. **d,** Schematic of processing pipeline.

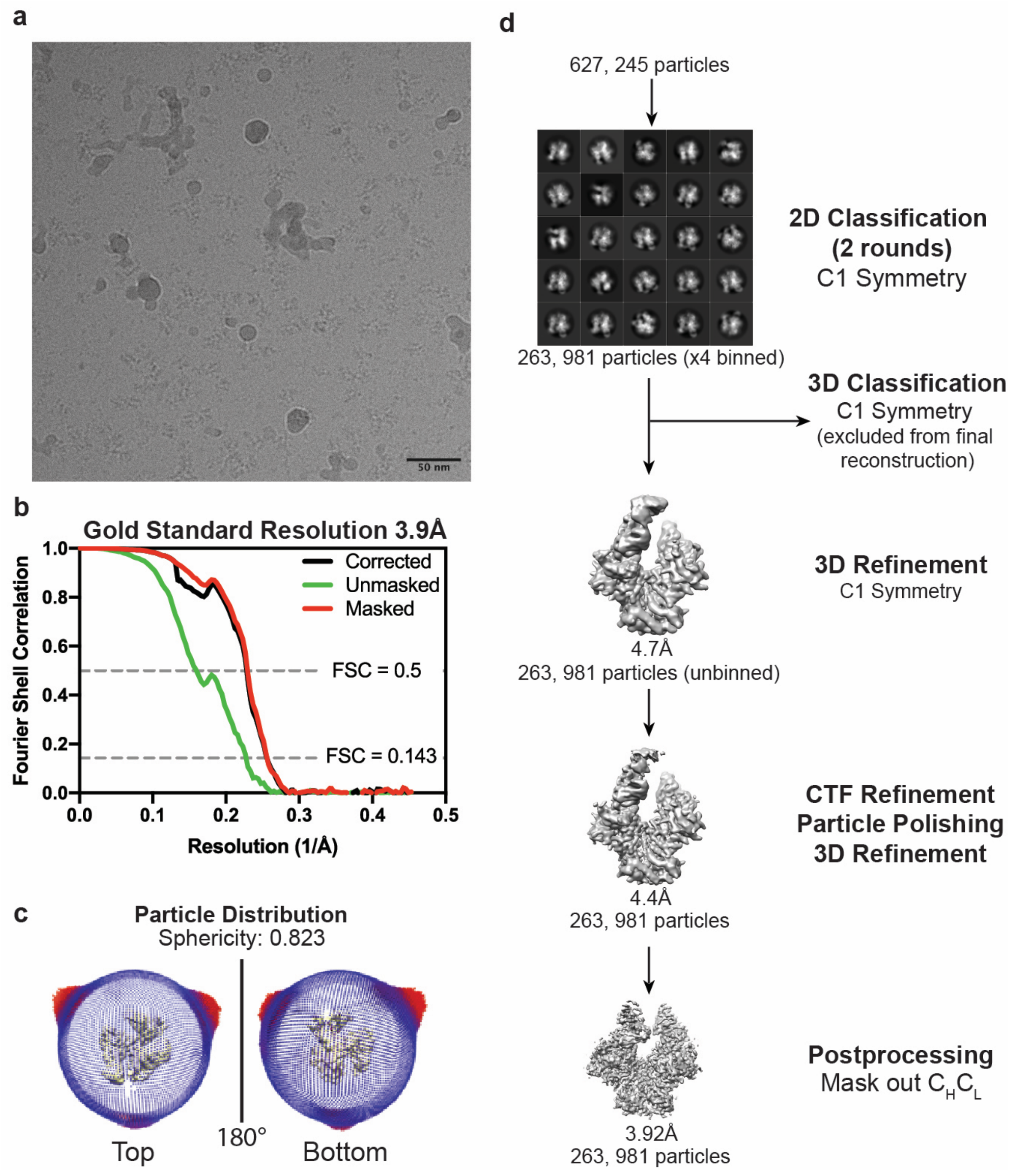

Supplementary Figure 3. Data Processing for the M48U1-BG505-17b complex.
a, Representative EM micrograph of data set. 2688 total micrographs were collected. Scale bar is 50nm. **b,** Gold Standard 3D FSC chart for final reconstruction map using combined data sets. **c,** Particle orientation distribution and sphericity for final reconstruction of combined data sets. **d,** Schematic of Processing pipeline.

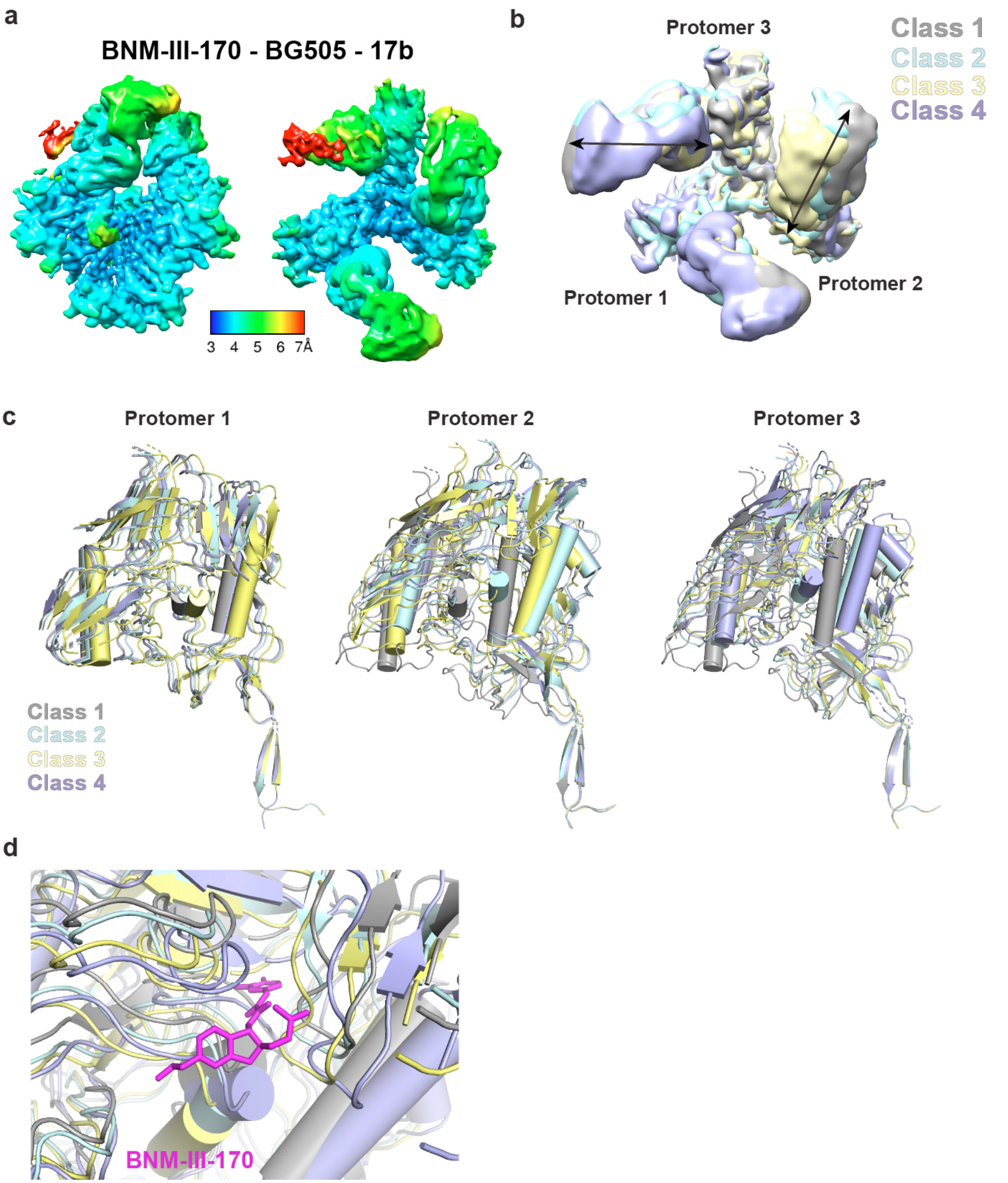

Supplementary Figure 4: 3D classification of BNM-III-170-BG505-17b shows differences in positioning of gp120 and 17b.

a, Local resolution map of BNM-III-170-BG505-17b reconstruction. **b,** Overlay of 3D classes of BNM-III-170-BG505-17b produced in final classification round after merging and polishing particles for classes (gray, pale cyan, pale yellow, light purple for Class 1-4, respectively). Double-headed arrow on Protomer 3 shows direction of displacement of 17b and gp120 between 3D classes. **c,** Cartoon models of gp120 subunits rigid body fit into BNM-III-170-BG505-17b 3D

classification maps. The β4/β26 strands were fit separately from the rest of the gp120 and alignments were done using Cα of β4/β26 strands. **d,** Cartoon model overlay of Protomer 3 showing gp120 Phe43 pocket for each 3D class and BNM-III-170 (magenta) from final model.

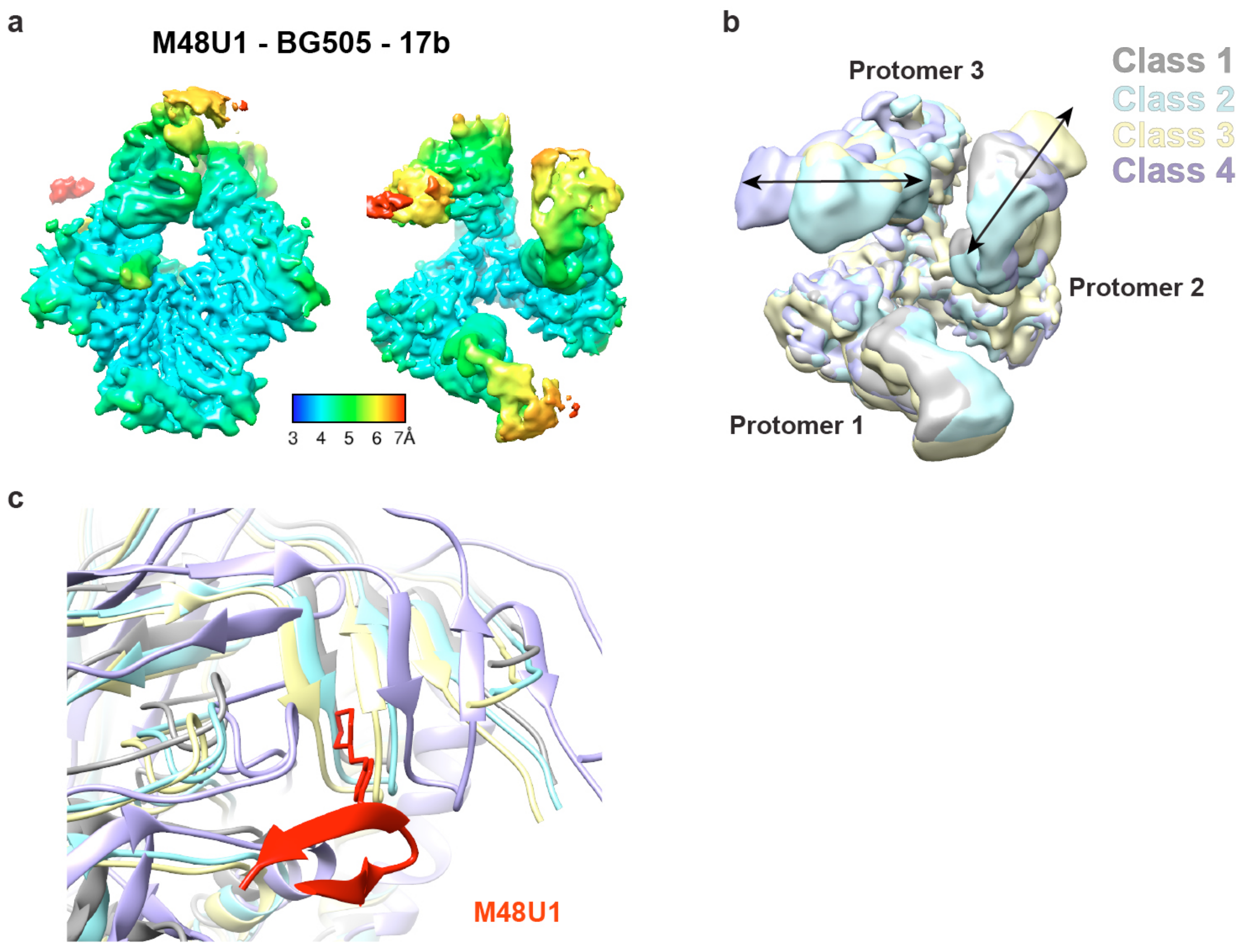

Supplementary Figure 5: 3D classification of M48U1-BG505-17b shows differences in positioning of gp120 and 17b.

a, Local resolution map of M48U1-17b reconstruction. **b,** Overlay of 3D classes of M48U1-BG505-17b (gray, pale cyan, pale yellow, light purple for Class 1-4, respectively). Double-headed arrow on Protomer 3 shows direction of displacement of 17b and gp120 between 3D classes. **c,** Cartoon model overlay of Protomer 3 gp120 Phe43 pocket for all 3D classes with M48U1 (red) from final model. M48U1 helix removed for clarity.

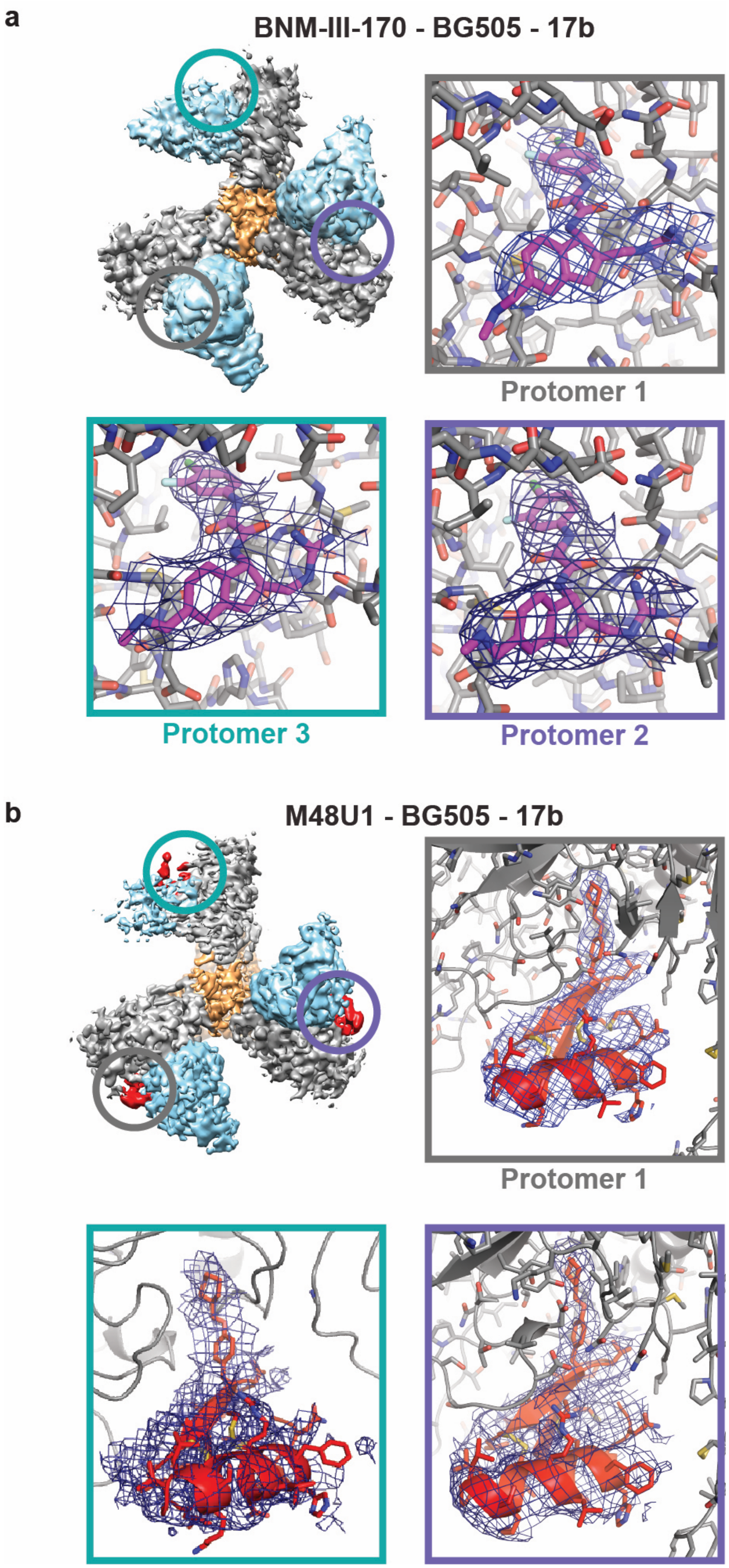
a
BNM-III-170 - BG505 - 17b
Protomer 1
Protomer 3
Protomer 2
b
M48U1 - BG505 - 17b
Protomer 1

Supplementary Figure 6. CD4m density is present within gp120 Phe43 cavity.

a, Top-down view of BNM-III-170-BG505-17b density (top left, 17b = light blue, gp120 = gray, gp41 = light orange) with regions containing BNM-III-170 circled. Surrounding panels show zoomed-in views of densities (blue) for the BNM-III-170 molecule and a cartoon/stick representation of the coordinates (BNM-III-170 = magenta, gp120 = gray) in Protomer 1 (gray), Protomer 2 (purple) and Protomer 3 (teal). Densities for protomers 1 and 2 are shown at 7σ and for protomer 3 at 5σ. **b**, Top-down view of M48U1-BG505-17b density (top left, 17b = light blue, gp120 = gray, gp41 = light orange) with regions containing M48U1 (red) circled in Protomer 1 (gray), Protomer 2 (purple) and Protomer 3 (teal). Surrounding panels show zoomed-in views of densities (blue) for the M48U1 molecule and a cartoon/stick representation of the coordinates (M48U1 = red, gp120 = gray) in each protomer. Density for protomer 1 is shown at 7σ and for protomers 2 and 3 at 5σ.

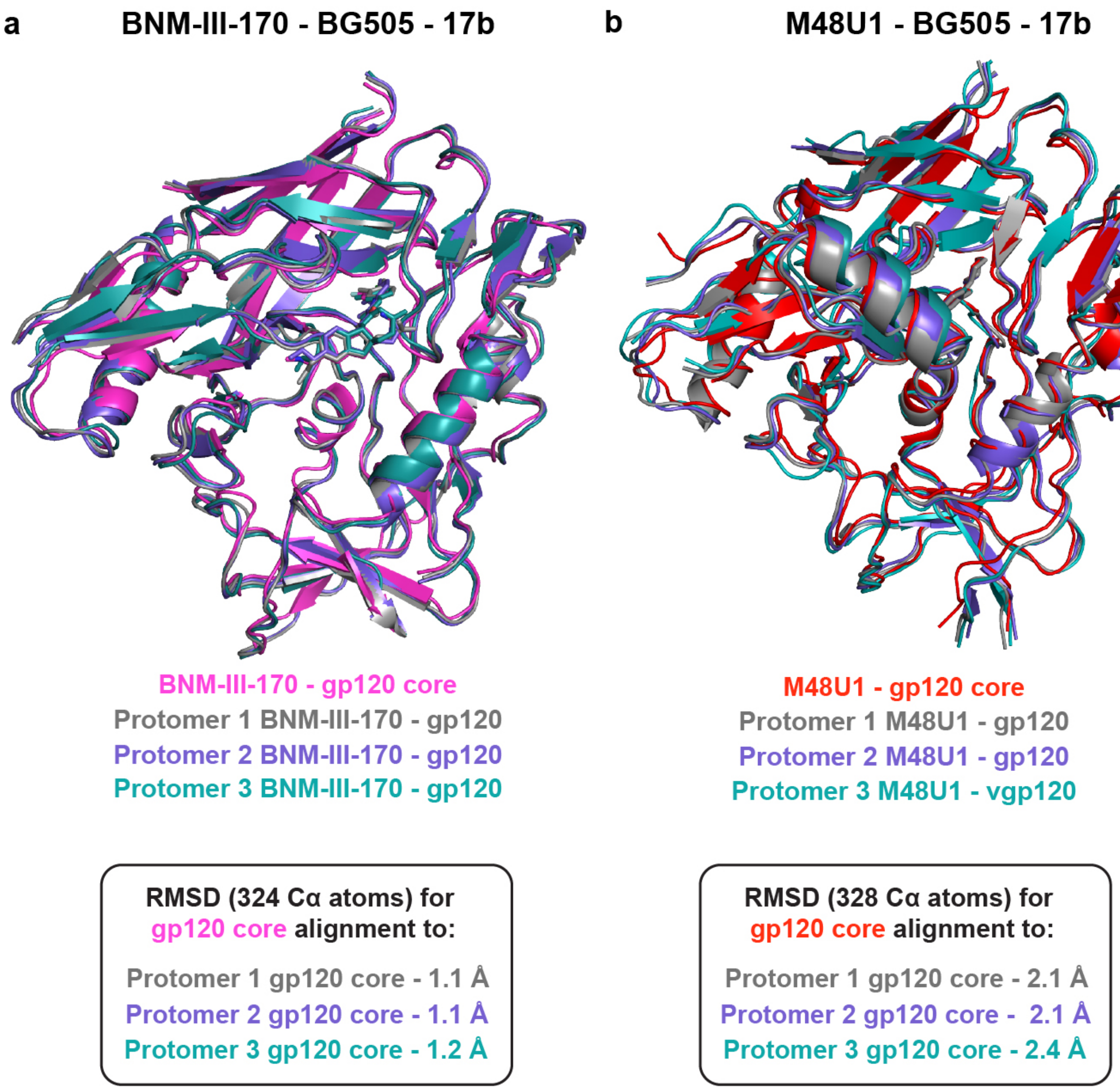

Supplementary Figure 7. Overlays of CD4m-gp120 core crystal structures with gp120 core portions of structures of CD4m-BG505 Env trimer complexes.
a, Alignment of BNM-III-170-gp120 core crystal structure (PDB 5F4P [https://doi.org/10.2210/pdb5F4P/pdb], magenta) and gp120 core regions from protomers 1 (gray), 2 (purple), and 3 (teal) of the BNM-III-170-BG505-17b complex**.** **b,** Alignment of M48U1-gp120 core crystal structure (PDB 4JZZ [https://doi.org/10.2210/pdb4JZZ/pdb], red) and gp120 core regions from protomers 1 (gray), 2 (purple), and 3 (teal) of M48U1-BG505-17b complex**.**

Supplementary Table 1: Cryo-EM data collection, refinement and validation statistics

	BNM-III-170-<br>BG505 SOSIP.664-17b<br>(Data Set 1)	BNM-III-170-<br>BG505 SOSIP.664-17b<br>(Data Set 2)	BNM-III-170-<br>BG505 SOSIP.664-17b<br>Combined Data<br>(EMD-23462)<br>(PDB 7LO6)	M48U1-<br>BG505 SOSIP.664-17b<br><br>(EMD-23465)<br>(PDB 7LOK)
**Data collection and processing**				
Magnification	81, 000x	81, 000x	81, 000x	81, 000x
Voltage (kV)	300	300	300	300
Electron exposure (e–/Å^2)	40	40	40	60
Defocus range (μm)	-1.5 to -3.5	-1.5 to -3.5	-1.5 to -3.5	-1.5 to -3.5
Pixel size (Å)	1.104	1.104	1.104	1.104
Symmetry imposed	C1	C1	C1	C1
Initial particle images (no.)	1040973	457694	n/a	627245
Final particle images (no.)	505426	158691	662861	263981
Map resolution (Å)	4.0	4.6	3.7	3.9
FSC threshold	0.143	0.143	0.143	0.143
Map resolution range (Å)	n/a	n/a	3.7 – 4.1	3.9 – 4.4
**Refinement**				
Initial model used (PDB code)	n/a	n/a	6U0L, 5F4P, 2NXY	6U0L, 4JZZ, 2NXY
Model resolution (Å)	n/a	n/a	3.7	3.9
FSC threshold			0.143	0.143
Model resolution range (Å)	n/a	n/a	3.67 – 3.8	3.8 – 4.0
Map sharpening *B* factor (Å^2)	n/a	n/a	-124	-150
Model composition				
Non-hydrogen atoms	n/a	n/a	18015	14498
Protein residues	n/a	n/a	2198	1999
Ligands	n/a	n/a	MAN: 9<br>BMA: 3<br>NAG: 47<br>5VG: 3	MPT: 3<br>BMA : 3<br>NAG: 14<br>NH2: 3<br>MAN: 6<br>DPR: 3
B factors (Å^2)				
Protein	n/a	n/a	144.19	219.21
Ligand	n/a	n/a	142.22	118.36
R.m.s. deviations				
Bond lengths (Å)	n/a	n/a	0.011	0.007
Bond angles (°)	n/a	n/a	0.944	1.132
Validation				
MolProbity score	n/a	n/a	2.40	2.27
Clashscore	n/a	n/a	19.03	14.44
Poor rotamers (%)	n/a	n/a	0.69	0.39
Ramachandran plot				
Favored (%)	n/a	n/a	87.08	87.99
Allowed (%)	n/a	n/a	12.87	11.85
Disallowed (%)	n/a	n/a	0.05	0.16
