# Supplementary material for: Cryo-EM structures of HIV-1 trimer bound to CD4-mimetics BNM-III-170 and M48U1 adopt a CD4-bound open conformation
Source: Nat Commun. 2021 Mar 29;12:1950. doi: 10.1038/s41467-021-21816-x (PMC8007822; doi:10.1038/s41467-021-21816-x)
Supplement: Supplementary file 3 — Reporting Summary [file 41467_2021_21816_MOESM3_ESM.pdf]

nature research

Corresponding author(s): Pamela Bjorkman

Last updated by author(s): 2/9/2021

Reporting Summary

Nature Research wishes to improve the reproducibility of the work that we publish. This form provides structure for consistency and transparency in reporting. For further information on Nature Research policies, see our Editorial Policies and the Editorial Policy Checklist.

Statistics

For all statistical analyses, confirm that the following items are present in the figure legend, table legend, main text, or Methods section.

n/a	Confirmed	
☐	☒	The exact sample size (*n*) for each experimental group/condition, given as a discrete number and unit of measurement
☐	☒	A statement on whether measurements were taken from distinct samples or whether the same sample was measured repeatedly
☒	☐	The statistical test(s) used AND whether they are one- or two-sided<br>*Only common tests should be described solely by name; describe more complex techniques in the Methods section.*
☒	☐	A description of all covariates tested
☒	☐	A description of any assumptions or corrections, such as tests of normality and adjustment for multiple comparisons
☐	☒	A full description of the statistical parameters including central tendency (e.g. means) or other basic estimates (e.g. regression coefficient) AND variation (e.g. standard deviation) or associated estimates of uncertainty (e.g. confidence intervals)
☒	☐	For null hypothesis testing, the test statistic (e.g. *F*, *t*, *r*) with confidence intervals, effect sizes, degrees of freedom and *P* value noted<br>*Give P values as exact values whenever suitable.*
☒	☐	For Bayesian analysis, information on the choice of priors and Markov chain Monte Carlo settings
☒	☐	For hierarchical and complex designs, identification of the appropriate level for tests and full reporting of outcomes
☒	☐	Estimates of effect sizes (e.g. Cohen's *d*, Pearson's *r*), indicating how they were calculated

Our web collection on statistics for biologists contains articles on many of the points above.

Software and code

Policy information about availability of computer code

Data collection	SerialEM data acquisition software (v3.8 Beta) was used for cryo-EM data collection of all data sets.
Data analysis	Phenix v1.17, Coot v0.8.9.1, RELION v3.0, Gctf v1.06, PyMOL v2.3.5, Chimera v1.14, Molprobity v4.4, CryoSPARC v2.15.0, 3DFSC server (https://3dfsc.salk.edu/) were used for structural analysis.

For manuscripts utilizing custom algorithms or software that are central to the research but not yet described in published literature, software must be made available to editors and reviewers. We strongly encourage code deposition in a community repository (e.g. GitHub). See the Nature Research guidelines for submitting code & software for further information.

Data

Policy information about availability of data

All manuscripts must include a data availability statement. This statement should provide the following information, where applicable:

- Accession codes, unique identifiers, or web links for publicly available datasets
- A list of figures that have associated raw data
- A description of any restrictions on data availability

Cryo-EM reconstructions of the BNM-III-170-BG505-17b and M48U1-BG505-17b complexes have been deposited in the Electron Microscopy Data Bank under the accession numbers EMD-23462 and EMD-23465, respectively. Coordinates for atomic models of the BNM-III-170-BG505-17b and M48U1-BG505-17b complexes have been deposited in the Protein Data Bank under the accession numbers 7LO6 and 7LOK, respectively.
Other data sets generated or analyzed during the current study are available from the corresponding author upon request.

Field-specific reporting

Please select the one below that is the best fit for your research. If you are not sure, read the appropriate sections before making your selection.

[x] Life sciences   [ ] Behavioural & social sciences   [ ] Ecological, evolutionary & environmental sciences

For a reference copy of the document with all sections, see nature.com/documents/nr-reporting-summary-flat.pdf

Life sciences study design

All studies must disclose on these points even when the disclosure is negative.

Sample size	not applicable to this study
Data exclusions	not applicable to this study
Replication	not applicable to this study
Randomization	not applicable to this study
Blinding	not applicable to this study

Reporting for specific materials, systems and methods

We require information from authors about some types of materials, experimental systems and methods used in many studies. Here, indicate whether each material, system or method listed is relevant to your study. If you are not sure if a list item applies to your research, read the appropriate section before selecting a response.

Materials & experimental systems

n/a	Involved in the study	
[ ]	[x]	Antibodies
[ ]	[x]	Eukaryotic cell lines
[x]	[ ]	Palaeontology and archaeology
[x]	[ ]	Animals and other organisms
[x]	[ ]	Human research participants
[x]	[ ]	Clinical data
[x]	[ ]	Dual use research of concern

Methods

n/a	Involved in the study	
[x]	[ ]	ChIP-seq
[x]	[ ]	Flow cytometry
[x]	[ ]	MRI-based neuroimaging

Antibodies

Antibodies used	17b IgG, 17b Fab, 10-1074 IgG, 21c IgG, BG1 IgG, PG16 IgG, JR-52 IgG
Validation	All antibody constructs were validated by sequencing. All antibodies in this study were expressed and purified in the Bjorkman Lab.

Eukaryotic cell lines

Policy information about cell lines

Cell line source(s)	Expi293F (Gibco)
Authentication	Cell lines were obtained from and authenticated by vendors or scientific collaborators.
Mycoplasma contamination	The cell lines were not contaminated by mycoplasma as determined by using the Lonza Mycoplasma Detection Kit.
Commonly misidentified lines (See ICLAC register)	None
